# Obituary

**Published:** 1896-01

**Authors:** 


OBITUARY.—Dr. W. U. Custer, of Reading, Pa., died suddenly in his office on the evening of December 1st, at the age of-sixty-five.

Dr. Custer was not a graduate of any college, but had followed his calling faithfully for more than a score of years and won for himself the good-will and appreciation of the community in which he lived. He was a member of the Pennsylvania State Veterinary Medical Association, and one of its most earnest supporters and regular attendants.

